# Editorial: Equitable digital medicine and home health care

**DOI:** 10.3389/fpubh.2023.1251154

**Published:** 2023-12-12

**Authors:** Francesco De Micco, Anna De Benedictis, Emanuele Lettieri, Vittoradolfo Tambone

**Affiliations:** ^1^Research Unit of Bioethics and Humanities, Department of Medicine and Surgery, Università Campus Bio-Medico di Roma, Roma, Italy; ^2^Fondazione Policlinico Universitario Campus Bio-Medico, Roma, Italy; ^3^Research Unit of Nursing Science, Department of Medicine and Surgery, Università Campus Bio-Medico di Roma, Roma, Italy; ^4^Department of Management, Economics and Industrial Engineering, Politecnico di Milano, Milan, Italy

**Keywords:** digital medicine, home healthcare, public health ethics, clinical risk management, policy and practice

Digital health technology (DHT) concerns the use of high-quality hardware and software to support medical practice, including diagnosis, treatment, disease prevention and health promotion for individuals and populations (1). DHT has made great progress in the last decade, radically transforming the way health care is delivered. Thanks to DHT, health can be monitored, diagnosed and treated in innovative and efficient ways (2, 3). Digital technologies are used to control asthma and COPD (4), to treat ADHD (5), to improve sleep quality (6), to treat low back pain (7), to manage type 2 diabetes (8), for improving executive function and emotional adjustment in the rehabilitation of traumatic brain injury (9) for monitoring substance use disorders (10) and for the early detection of acute kidney injury (AKI) (Shi et al.). This non-exhaustive list of digital therapies shows the wide, varied and hypothetically boundless spectrum in which the development and use of DHT can implement health.

Then, there was the COVID-19 pandemic that posed unprecedented challenges to the global health system, highlighting the importance of home care and the role that DHT can play in a context where “moving information” is much better than “moving people” (11).

During the pandemic emergency, there was a significant increase in investment in digital health technology solutions, with a record USD 24 billion investment in digital health in 2020, and a new monthly record in December 2020 of USD 3.4 billion (12).

Digital technologies have been crucial in the fight against coronavirus because they have enabled contact tracing (13), monitoring (14) and diagnosis (15) of citizens and patients during a global pandemic in which without social distancing there would have been a progressive and uncontrolled spread of the virus.

As the world adapts to the “new normal”, home care has undergone a significant evolution from a complementary service to a primary form of care (16). DHT is joining home care to create a powerful combination, bringing innovation, efficiency and equitable access to healthcare into the homes of millions of people around the world. This synergy is revolutionizing the way healthcare is delivered, enabling people to receive appropriate and personalized treatment in their own homes. Estebanez-Pérez et al. show how in rural areas of India, DHT makes it possible to overcome the shortage of physiotherapists and ensure the rehabilitation of children with ankle fractures by improving functional independence and quality of life.

However, access to these digital health solutions is not equally distributed throughout the world. DHT is more developed in urban areas than in rural areas, is used more by young, white, English-speaking individuals with higher education and higher economic status. Moreover, paradoxically, better access to DHT has been found in individuals without disabilities or complex health needs (17). In this scenario, in order to optimize potential and concrete improvements in health care, it is necessary to overcome certain limitations, well-highlighted by the study of Cingolani et al.: the shortcomings of health information systems and digital tools, the slow spread of electronic medical records, the problems of digital literacy, the high cost of devices, and the poor protection of data privacy. The danger of over-reliance on such systems must also be examined.

The political decision-makers of each country have the fundamental responsibility to establish and ensure a robust legal and regulatory framework that places the protection of patients at the center within the healthcare context. This legal framework should clearly outline patients' rights, including equitable access to care, privacy, and the security of medical data, as well as mechanisms for recourse in the event of medical errors or violations of patients' rights (Cingolani et al.). The clarity and robustness of the legal framework are essential to ensure patients' trust in the healthcare system and to guarantee that they are treated fairly, safely, and in a manner that respects their rights. Furthermore, a sturdy legal foundation promotes accountability among healthcare professionals and institutions involved in delivering care, thereby contributing to enhancing the overall quality and safety of healthcare services (18).

Home care based on equitable digital medicine aims to overcome this inequality and ensure that everyone, regardless of their geographical location or socio-economic background, can benefit from advances in DHT.

DHT must be a means through which all citizens have equal access to healthcare. Thanks to DHT, patients can be monitored, diagnosed and treated remotely, eliminating the need to physically travel to the hospital. This is especially relevant for people who live in remote areas or who have mobility difficulties, enabling them to receive appropriate care without having to make long and expensive journeys (19).

Among digital technologies applied to healthcare, telemedicine is a key pillar for equitable DHT in home care. Telemedicine can contribute to a reorganization of health services, enabling the shift of health care from the hospital to the local area, through innovative patient-centered care models and facilitating access to services for people who would otherwise have difficulty traveling to a traditional health facility, such as the older adult, the disabled or those living in rural areas (20–24). However, Bashir et al. showed that successful telemedicine requires the creation of specialized educational programs for healthcare workers (HWs) to ensure proper implementation.

Another key to equitable DHT in home care is remote monitoring and the use of wearable devices. Thanks to advanced sensors and devices such as smartwatches or smart bracelets, patients can measure their vital parameters, monitor physical activity, check glucose levels or track other important indicators of their health. This data is sent in real time to HWs who can carry out constant monitoring and intervene promptly in the event of anomalies (25–29). This allows personalized treatment and more accurate monitoring of the patient's health condition as well as increasing patient empowerment. Through the use of mobile applications, patients can access health information, educate themselves about medical conditions and manage their own wellbeing more independently. This increased awareness and responsibility for one's own health enables patients to make informed decisions and actively participate in the treatment of their illnesses (30). In addition, data analysis can allow the identification of potential risks, enabling early intervention to prevent the development of diseases. This proactive approach to health could help reduce the incidence of chronic diseases and improve people's quality of life.

In order for everyone to benefit from DHT-based home healthcare, it is necessary for policy-makers to take up and address important challenges.

a) Establish regulations, develop guidelines with methodological rigor, quality, transparency and accuracy while reducing the risk of bias (Silva et al.) and formulate guidelines to support decision-making in digital health (17). According to Petrini et al., special regulatory attention should also be paid with regard to decentralized clinical trials (DCTs) that rely on the use of digital tools such as electronic consent, apps, wearable devices, electronic patient reported outcomes (ePROs), telemedicine, as well as on moving trial activities to the patient's home.
b) Less than half of the population in developing countries has access to the internet. Advanced economies such as the US, France, Germany, the UK and Canada have the highest access rates. The large emerging economies show large disparities in the proportion of Internet users in their population, ranging from about two-thirds in Brazil and Mexico to about one-third in India (31). Without reliable connectivity and appropriate devices, it becomes difficult for people to fully benefit from digital health services. Therefore, efforts by governments and international organizations are needed to improve access to the technology infrastructure and promote global connectivity.
c) DHT “feeds” on personal information that is collected and shared. This is the essence of DHT. Therefore, it is crucial to ensure that patient data are properly protected and only used for legitimate purposes. Furthermore, it is mandatory to take appropriate and proportionate technical and organizational measures to manage risks and to prevent and minimize the impact of network and information system security incidents. Patient safety should be supported by governance activities that strengthen the information infrastructure (Verga et al.).
d) DHT must be designed considering the different cultural backgrounds and specific needs of local communities. This involves the translation of medical applications and content into local languages, as well as the adaptation of medical practices to specific cultural contexts. In this respect, Kim's study is very interesting: it is undeniable that the use of ITC can be beneficial for the health care brought to the older adult. But this is not enough, because there is a need for consistency between national health policies and the choice to use technological devices to manage the health of the older adult (Kim).
e) The adoption of digital technologies in healthcare can depersonalize and negatively affect the care relationship leading to insufficient communication and limited data transmission that can expose the patient to clinical risk in multiple situations (32, 33). For this reason, home care supported by DHT cannot completely replace the need for traditional health care. DHT and home care should be integrated into a holistic approach to health care that considers the individual needs and circumstances of patients.

Equitable digital medicine has the “potential” to revolutionize access to health care worldwide. However, for it not to remain only a “potential” benefit for a large part of the world's population, geographic and economic barriers must be overcome and health services must be offered to everyone who needs them. New legislation, targeted investment in technology infrastructure, data protection policies and an inclusive approach can certainly help to create a future where everyone has access to high quality healthcare. Doing the right thing for the right patient at the right time requires that all interventions are based on a strong ethical framework. The patient's centrality, the medical act as a free and responsible human act with an intrinsic ethical value, interdisciplinary co-design, a realist knowledge, a management model based on motivational involvement, professional excellence as an instrument of service to society and the common good, and the capacity for radical procedural innovation are the pillars of the ethics of a job well-done (34) and could constitute the ethical guidelines on which to build an equitable DHT serving all citizens, especially the older adult, the disabled or those living in remote areas (35).

## Author contributions

VT and FD wrote the first version of the manuscript. All authors made a significant contribution to this paper and have read and approved the final version of the manuscript.
